# Coverage of the scrotum after Fournier’s gangrene

**DOI:** 10.3205/iprs000121

**Published:** 2018-01-15

**Authors:** Ahmed Hassan El-Sabbagh

**Affiliations:** 1 Plastic Surgery Center, Faculty of Medicine, Mansoura University, Egypt

**Keywords:** Fournier's gangrene, scrotum, scrotal advancement flap, pudendal thigh flap

## Abstract

**Background:** Fournier’s gangrene is a necrotizing fasciitis caused by mixed aerobic and anaerobic bacteria and results in loss of skin and subcutaneous tissue in the perineal area. Coverage of testis varies from closure of the defect primarily, burying inside the thigh, using the remnants of the scrotum for tissue expansion and coverage by flaps. In this manuscript, scrotal advancement flaps and pudendal thigh flaps were used for coverage of the testis unilaterally or bilaterally according to the size of the defect following Fournier gangrene.

**Patients and methods:** From June 2015 to March 2017, twelve cases were admitted to our department. The patients’ ages ranged from 37–59 years and they all had suffered from Fournier’s gangrene in the perineal area.

**Results:** Of the twelve cases, two cases showed penile involvement. A skin graft was used for coverage of the penile shaft with excellent take. Four cases were closed primarily. This was applied to cases where loss of skin was less than 50%. The rest of the cases were reconstructed by pudendal thigh flap. The reconstructed cases were covered by bilateral pudendal thigh flap (4 cases) and unilateral pudendal thigh flap (4 cases). The follow-up extended up to 16 months.

**Conclusion:** Scrotal advancement flap was suitable for small and medium size defects due to the elasticity of the scrotal skin. Pudendal thigh flap was efficient for the reconstruction of large defects of the scrotum.

## Introduction

Since the first description of Fournier's gangrene, it is still considered a disastrous clinical condition. Several methods have been described for its management and reconstruction [1].

Fournier’s gangrene is a necrotizing fasciitis, caused by infecting organisms and results in loss of skin and subcutaneous tissue in the perineal area. It is more common in patients with diabetes mellitus, impaired immunity, alcoholism, inflammatory anorectal diseases, urinary incontinence, and overall debilitating diseases [2]. It requires early diagnosis and aggressive debridement to remove all dead tissues. The necrosis involves the scrotum and does not include the testis and/or spermatic cord [3]. However, the exposure of the testis can cause severe functional, aesthetic and psychological harm to the patient. 

Coverage of the scrotum varies from closure of the defect primarily, orchiectomy with spontaneous closure, burying the testes inside the thigh and using the remnants of the scrotum for tissue expansion [4]. Also, flaps from the thigh can be used. 

Requirements for successful scrotal reconstruction include an esthetically acceptable appearance of the scrotum, relatively hairless coverage, durable reconstruction, hidden donor site, with good physiological function of the testis. 

Herewith, scrotal advancement flap and pudendal flaps were used for coverage of the scrotum after Fournier’s gangrene.

## Patients and methods

### Patients

From June 2015 to March 2017, twelve cases were admitted to our department. Consents were obtained from all patients involved in this series. Separate consents were taken for photography. The patients’ ages ranged from 37–59 years and they all had suffered from Fournier’s gangrene in the perineal area. Common predisposing factors were diabetes mellitus, chronic renal failure, and lack of personal hygiene. The patients were referred from the general surgery department. 

### Operative technique

#### Arterial supply

The *internal pudendal artery* is a terminal branch of the anterior division of the internal iliac and is the blood supply of the perineum. It passes subcutaneously to a triangular area formed by ischial tuberosity, urethral opening, and the anus. It continues as the posterior scrotal artery which divides into two to three branches supplying the scrotum [5]. 

The *external pudendal artery* arises from the medial side of the femoral artery and divides into the superficial and deep branches. It sends cutaneous branches that supply the skin of the femoral triangle and travels medially. It terminates inferiorly below symphysis pubic to supply the skin of the scrotum anteriorly [6].

The *cutaneous branches of the obturator artery* arborize medially near the origin of gracilis muscle. It gives cutaneous branches that supply the skin of the middle third of the scrotum [7].

Anastomoses occur between the branches of the internal pudendal, the external pudendal and the obturator arteries. So, the pudendal thigh flap can be raised over the three vascular territories of the three arteries (urogenital triangle). As regarding nerve supply, the path of the superficial perineal nerve accompanies the branches of the superficial perineal vessels [8].

#### Scrotal advancement flap

The first step was the surgical refreshment of the wound and the removal of any granulation tissues. If both testes were involved, they were sutured together. If less than 50% of the scrotum was lost, a scrotal advancement flap was made. The flap was elevated in a plane close to the tunica vaginalis to protect the main vessels and the wound was closed primarily.

#### Pudendal thigh flap 

**Figure 1:**
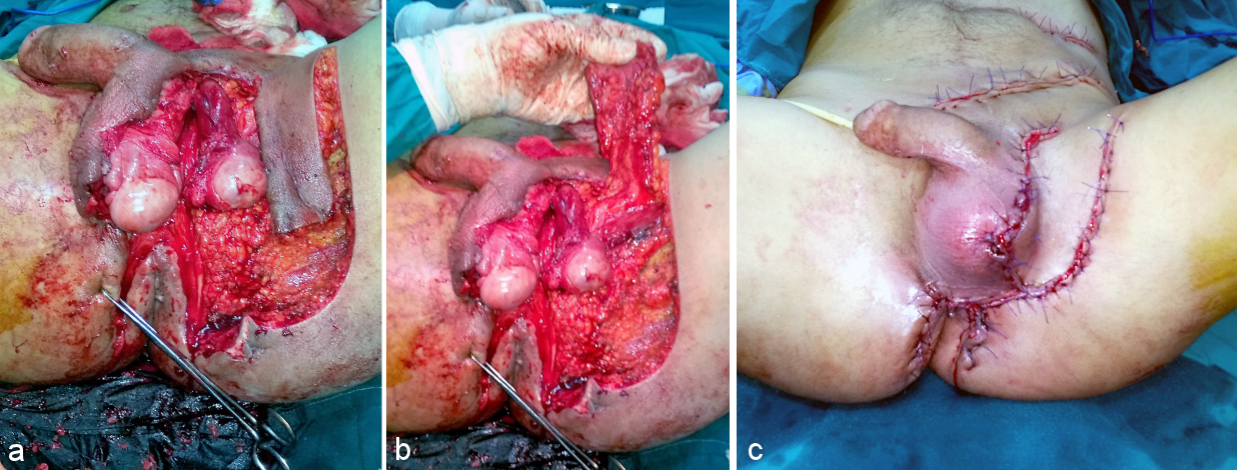
Unilateral pudendal thigh flap. a: Designing the flap. b: Undersurface of the flap. c: Postoperative view.

The flap was based on cutaneous branches of external pudendal artery (Figure 1 (Fig. 1)). A Doppler was used to identify vessels (Attachment 1).

The incisions were taken down to the deep fascia on both sides. Tacking sutures were taken between deep fascia and edges of skin. The deep fascia was sutured to the skin edges. The distal margin of the flap was incised and the flap was elevated as a fasciocutaneous flap. The flap was based over the superior edge of the adductor longus muscle with inclusion of the deep fascia and the epimysium of muscle. The size of the flap was adjusted to the size of the defect and the length of the required flap. Up to 14 × 8 cm flap size was achieved. The drains were inserted at the donor sites. 

## Results

**Figure 2:**
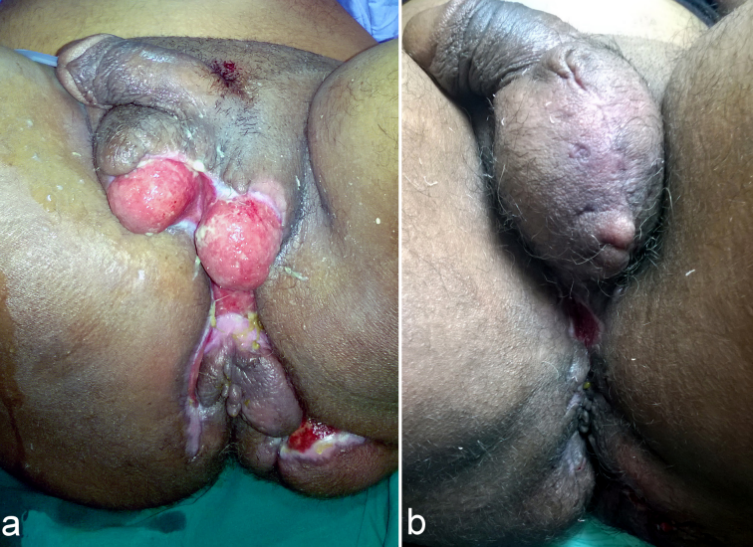
Partial coverage of both testes treated by scrotal advancement flap. a: Preoperative view. b: Postoperative view (after 1 month).

**Figure 3:**
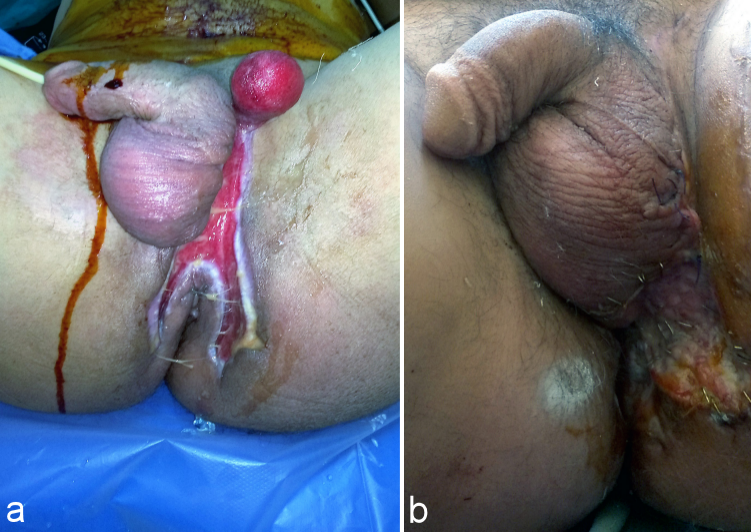
Complete exposure of the left testis and partial coverage of the right testis treated by unilateral pudendal thigh flap. a: Preoperative view. b: Postoperative view (after 2 months).

**Figure 4:**
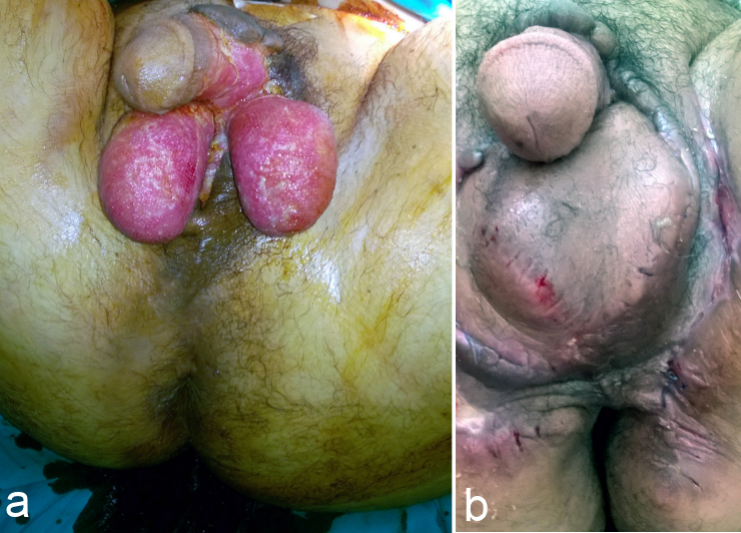
Complete exposure of both testes and the shaft of the penis treated by bilateral pudendal thigh flaps and skin graft to the shaft of the penis. a: Preoperative view. b: Postoperative view (after 2 months).

Of the twelve cases, two cases showed penile involvement. Skin graft was used for coverage of the penile shaft with excellent take. Four cases were closed primarily by scrotal advancement flaps where loss of scrotum was less than 50% (Figure 2 (Fig. 2)). The rest of the cases were reconstructed by pudendal thigh flaps (8 cases). Half of these cases were reconstructed by a unilateral pudendal thigh flap and the other half by a bilateral pudendal thigh flap (Figure 3 (Fig. 3), Figure 4 (Fig. 4)). The follow-up extended up to 16 months (Table 1 (Tab. 1)).

Small wound dehiscence occurred in two cases. One case showed dehiscence between scrotal and anal area after scrotal advancement flap. The other case showed dehiscence at the distal end of the donor site of the flap. In the following cases, any areas under tension were covered with skin graft or left to heal by secondary intention. Distal flap necrosis occurred in one case. This may be due to an extension of the injured area to the territory of the flap. The case was managed conservatively with successful outcome. Seroma occurred at the donor site in one case. By frequent aspiration twice per week the problem was resolved in three weeks. None of the cases complained about donor sites scars.

### Case presentation 

**Figure 5:**
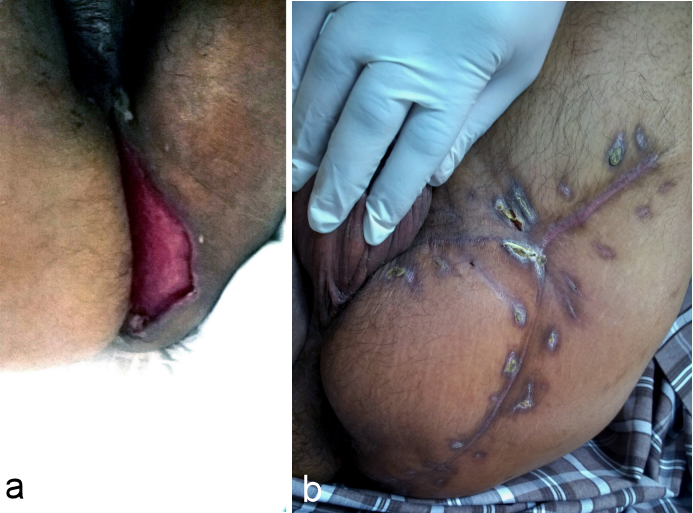
Defect in the perineal area treated by V-Y pudendal thigh flap. a: Preoperative view. b: Postoperative view (after 12 months).

A 66-year-old diabetic male patient suffered from necrotizing fasciitis in the perineal area (Figure 5 (Fig. 5)). He presented to our department with a full thickness defect on the left side of the perineum that was extended to the anal verge. Diabetes was tightly controlled and the wound was frequently dressed till healthy granulation tissue appeared in the injured area. A unilateral V-Y island fasciocutaneous flap was elevated based on cutaneous branches of the obturator artery. A Doppler was used for marking of the feeding vessels preoperatively. The size of the flap measured 19 × 12 cm. The healing was excellent with primary closure of the donor site. 

## Discussion

Baurienne and Hebler were the pioneers that made early description of Fournier’s gangrene. In 1883, Fournier mentioned the idiopathic origin of a rapidly fulminating genital gangrene in males. Later, in 1952, Wilson introduced the term necrotizing fasciitis [9].

The scrotal skin is elastic and stretchable. When less than 50% of skin of scrotum is lost, the scrotal advancement flap was a good option that was done in four cases. It has a good skin quality and an excellent aesthetic appearance [10]. In this work, one postoperative complication occurred due to closure under tension in the area between scrotum and anus. 

Burying the testis in the subcutaneous pouches in the thigh had been described [11]. This maneuver bears a lot of problems such as fainting attacks, testicular atrophy and sense of fullness in the thigh with unacceptable appearance.

Skin grafts were described for coverage of the testis. Healthy granulation tissue and intact tunica vaginalis are minimal requirements for a successful take. Although it has a good cosmetic result, it is liable for maceration and breakdown. During this work, skin grafts were used for coverage of the penile shaft in two patients giving acceptable results [12].

Negative wound therapy is relatively contraindicated in this area. It showed difficulty in maintaining the sealing of the sponge due to the presence of both urogenital and defecation structures. Weinfeld managed four cases using VAC therapy with successful coverage [13].

Scrotal defects are better reconstructed using local tissue because of their reliability and good matching with deficient tissues [14]. The extent of the defect and the orientation of the defect are critical for the determination of the required flap [15]. 

Wee and Joseph from Singapore had the credit for description of the pudendal thigh flap (Singapore flap) [16] which later was modified by Woods and his team and was used for surgical reconstruction of the vagina [17]. 

In this study, when there was complete loss of scrotal skin with exposure of both testes, the pudendal thigh flap was used bilaterally. Thanks to the elasticity of scrotal skin, presence of the remnant of scrotal skin on one testis was sufficient to use one unilateral pudendal thigh on the other side with complete coverage of both testes (8 cases). Complications after pudendal thigh flap were minor. Small wound dehiscence (one case), seroma (one case) and distal flap necrosis (one case) occurred. All the complications were managed conservatively. 

The anterolateral thigh is used as a free flap for different areas of the body. When used as a local flap, it is successful for reconstruction of the scrotum [18]. However, medialization of the anterolateral thigh flap limits the arc of rotation and the pedicle length and consequently puts the flap under tension. Also, an anterolateral thigh flap due to its variable vascular supply needs a more tedious learning curve for dissection. In contrast, the pudendal thigh flap is relatively thin and the donor site is hidden and is closed primarily. Although one anterolateral thigh flap can substitute two pudendal thigh flaps for coverage of the two tests, this is considered as an advantage for pudendal thigh flap rather than a disadvantage. Closure of both flaps in midline preserves the midline line raphe with superior aesthetic results. In addition the use of single unilateral flap for total coverage of both testes may result in deviation of the new scrotum to one side with unaesthetic appearance.

The skin island of gracilis flap was used for perineal reconstruction [19]. However, the flap has several drawbacks. It has a high incidence of partial necrosis, the skin is thick and the scars on the thigh are ugly in appearance. In comparison, the pudendal thigh flap is relatively easy, the donor site scar is hidden and no muscle is scarified.

For successful reconstruction, six important points has to be addressed. First, the patient should have stable general condition with healthy granulation tissue of the wound before surgical interference. Second, surgical refreshing of the wound must be made preliminary before coverage. Third, both testes when exposed have to be sutured together to minimize tension on the flap and to decrease the size of the defect and consequently the size and number of flaps. Fourth, non-absorbable sutures are preferred for closure in this area to withstand the chemical effect of feces and urine till healing occurred. Fifth, do not close anything under tension. Lastly, drains have to be used to eliminate dead spaces and prevent fluid collection. 

A drawback of this study is the small number of patients in this study (12 patients). This is because all cases were confined to Fournier’s gangrene and were operated by one surgeon. 

**Figure 6:**
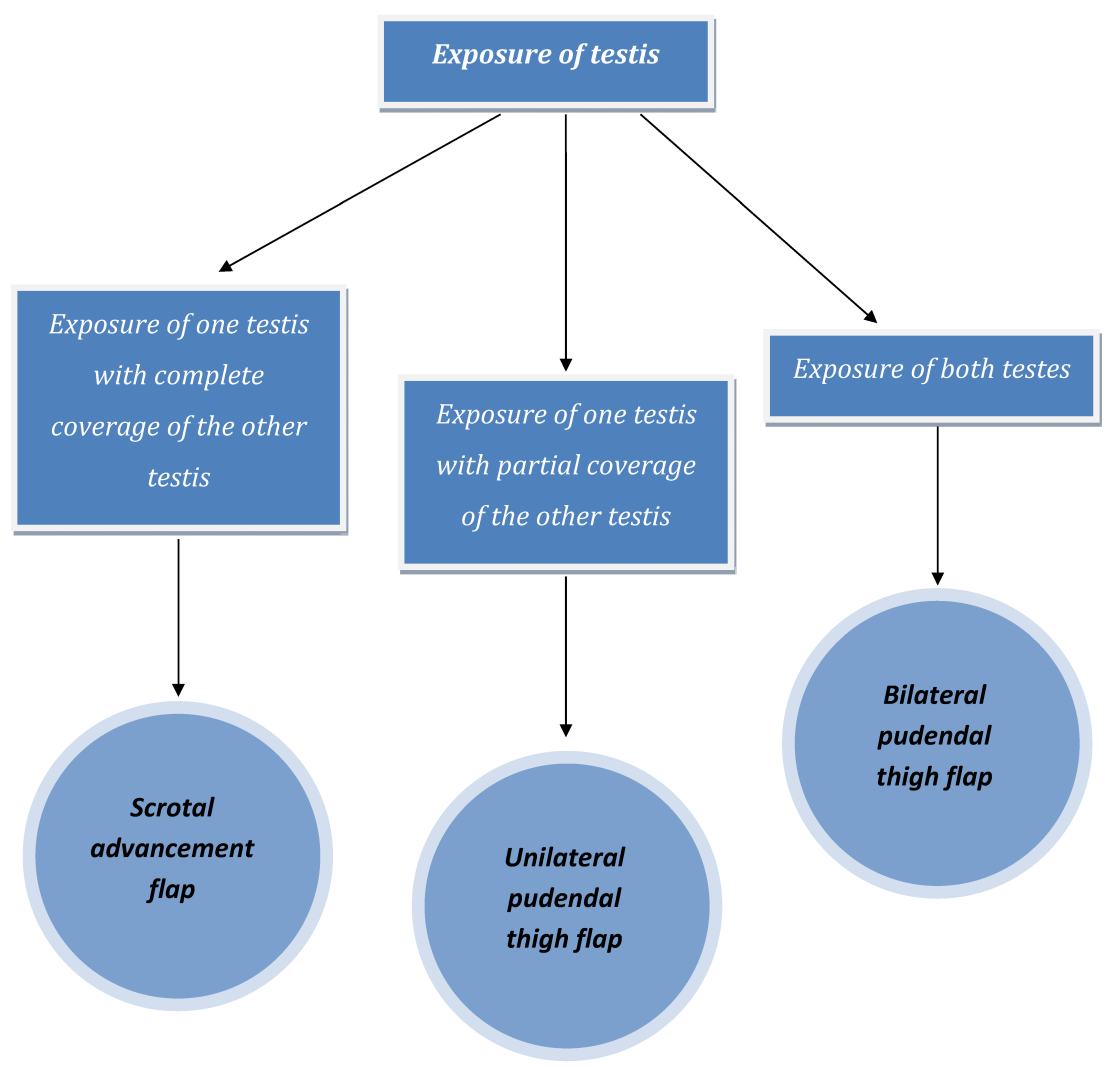
Algorithm for coverage of the scrotum

In summary, an algorithm was developed for the coverage of the scrotum (Figure 6 (Fig. 6)). Complete coverage of one testis with exposure of the other testis was sufficient to permit primary closure of both testes. However, partial coverage of one testis with exposure of the other testis was an indication for unilateral pudendal thigh flap. Bilateral pudendal thigh flaps were used when bilateral testes were exposed. Actually, pudendal thigh flap, it has a good vascular supply, relatively thin contour. Also, it is a sensate flap and has close proximity to the scrotum. In addition, it leaves a relatively inconspicuous donor site scar because of the coincidence with groin creases. All these criteria make the pudendal thigh flap superb in comparison to the use of skin grafts or other local flaps.

## Conclusion

The affection of genetalia carries special psychological insult as it is a representative sign of masculinity to many patients. In this work, the scrotal advancement flap was suitable for small and medium size defects of the scrotum. The pudendal thigh flap was a safe and dependable method for coverage of large defects of the scrotum. Flaps could be used unilaterally or bilaterally with little blood loss. A direct closure of the donor site was achieved in all cases with hidden donor site.

## Notes

### Competing interests

The authors declare that they have no competing interests.

### Ethical approval

Ethical approval for this kind of study did not require formal consent from a local ethics committee. 

### Informed consent

Informed consent was obtained from all patients.

## Supplementary Material

Video: Using Doppler to confirm the pedicle location

## Figures and Tables

**Table 1:**
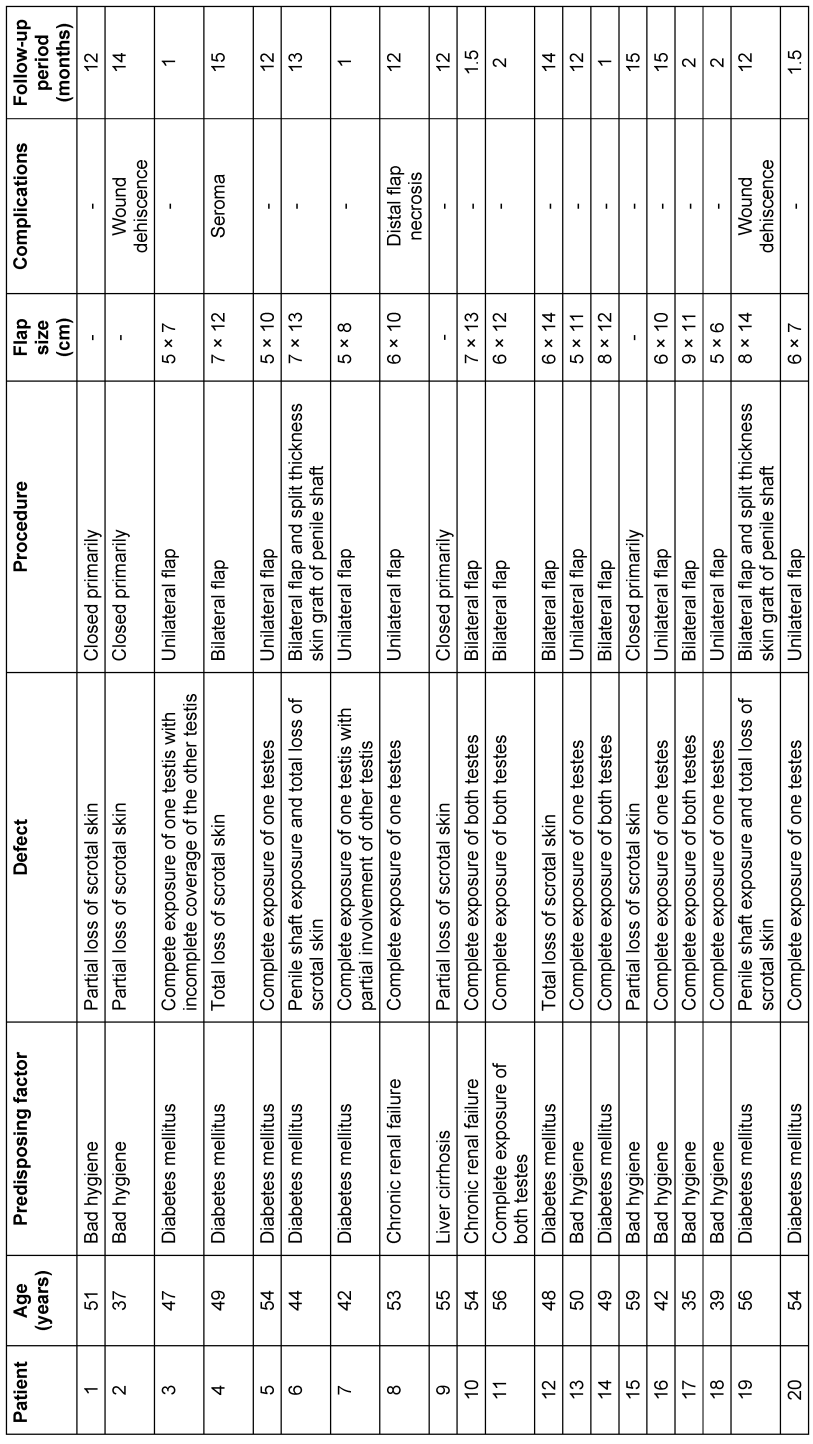
Summary of the results
